# A hospital-based study on clinical data, demographic data and visual function of keratoconus patients in Central China

**DOI:** 10.1038/s41598-021-87291-y

**Published:** 2021-04-06

**Authors:** Kaili Yang, Liyan Xu, Qi Fan, Yuwei Gu, Bo Zhang, Feiying Meng, Dongqing Zhao, Chenjiu Pang, Shengwei Ren

**Affiliations:** Henan Provincial People’s Hospital, Henan Eye Hospital, Henan Eye Institute, People’s Hospital of Zhengzhou University, Henan University People’s Hospital, 7 Weiwu Road, Zhengzhou, 450003 Henan People’s Republic of China

**Keywords:** Eye diseases, Health care

## Abstract

China is a populous country but lacks epidemiological data on keratoconus (KC). The present study aimed to investigate the clinical data, demographic data, and visual function (VF) data of KC patients in Central China. A total of 524 KC eyes in 307 KC patients (217 bilateral and 90 unilateral) from Henan Eye Hospital were included in the current study. Demographic and VF data were assessed with questionnaires administered by well-trained staff during face-to-face interviews. Visual acuity value was examined by a qualified optometrist, and the clinical data were measured by professional clinicians. The distributions of sex, residence and education level of KC patients were compared by Chi-square tests, and the ratios of people wearing glasses and rigid gas permeable (RGP) lenses were compared by McNemar tests. General linear models/Chi-squared tests were used to compare the clinical and demographic data according to KC severity. Spearman’s correlation analysis was used to test the associations between the data and KC severity. The mean age at diagnosis was 20.98 ± 6.06 years, and males had a higher ratio of KC than females (*P* < 0.001). Patients in rural areas had a higher rate of KC than those in urban areas (*P* = 0.039), and the proportion of KC patients with a higher education level (above high school) was high (*P* < 0.001). A total of 68.40% of the patients reported eye rubbing and 3.52% had a positive family history. The percentage of people wearing glasses was higher than that of patients wearing RGP lenses (*P* < 0.001). The total VF score of KC patients was 69.35 ± 15.25. The thinnest corneal thickness (TCT) and stiffness parameter at the first applanation (SP-A1) values were inversely correlated with KC severity (*P* < 0.05). The mean, steep, and max keratometry (Km, Ks and Kmax) values, the RGP lens use and keratoplasty were positively correlated with KC severity (all *P* < 0.05). The total VF score of the eye with better VA decreased as the severity increased (r = − 0.21, *P* = 0.002). The present study comprehensively describes various associated features of KC patients from a tertiary hospital in Central China, providing a reference for understanding the characteristics of KC patients in China.

## Introduction

Keratoconus (KC) is a corneal disorder characterized by corneal thinning, vision deterioration and irregular astigmatism that usually starts in the early teens^1^. The prevalence of KC was reported to be 1.38 per 1000 in a recent review^2^. KC etiology is currently unclear now, and the genetic and environmental factors have been reported to play important roles^3–6^. Epidemiological investigation of KC is helpful to further understand this condition.

The clinical findings of KC are mostly obtained through slit-lamp biomicroscopy, corneal topography, corneal tomography and corneal biomechanical examinations^7^. A previous study demonstrated that clinical measurements could provide references for evaluating the progression and disease severity of KC as well as treatment effects^8^. In addition, patients with KC experience ocular discomfort and poor visual acuity (VA), leading to impaired ability to perform social duties^9^. Mahdaviazad et al.^10^ found that the physical, emotional, and social functions were impaired in 30 Iranian KC patients. Yildiz et al.^11^ indicated that visual function (VF) in 149 Pennsylvania KC patients remained impaired after penetrating keratoplasty. In addition, Saunier et al.^12^ reported that KC patients subjectively perceive a loss of VF disproportionate to that reflected by clinical measurements. Thus, evaluating the clinical measurements and VF assists in comprehensive assessments of KC patients^13^.

KC patient characteristics have been investigated in previous studies worldwide; however, the results are controversial^5,14,15^. Khor et al.^5^ reported that KC patients in Asia had similar demographic and clinical characteristics to patients in Western populations. Despite being a populous country, China is a developing country, and research on Chinese KC patient characteristics is still limited. Xu et al.^16^ reported that the prevalence of steep cornea/KC was 0.9 ± 0.2% in people aged > 50 years who lived in Beijing, northern China, and the prevalence was associated with ocular parameters. However, the disease was defined based on only corneal refractive power, and the corneal topography that could detect minor forms of KC was not included in the study design. Jian et al.^17^ evaluated the ocular dimensions of KC eyes from Shanghai, eastern China, and found that KC eyes were characterized by deeper anterior chamber depths but shorter optical axial lengths and posterior segment lengths in adolescents (11–18 years). The demographic data, VF questionnaire data and corneal biomechanics of KC patients were not evaluated. In addition, Ma et al.^18^ found that corneal thickness in healthy eyes was significantly inconsistent among different regions, indicating that corneal parameters might not be consistent in different regions of China. Henan Province is located in Central China and is the third most populous province in China, with 96.4 million permanent residents in 2019. The province is dominated by agriculture, and the economic and medical conditions are different from those of other regions. There is a paucity of epidemiological research combining clinical and demographic data of KC patients in Central China. Thus, the present study aimed to investigate 307 KC patients’ characteristics from a hospital-based population, combining clinical data, demographic data and VF questionnaire responses.

## Methods

### Study subjects

KC patients were consecutively enrolled in this descriptive study from June 2018 to January 2020 at Henan Eye Hospital & Henan Eye Institute. The hospital is one of the earliest ophthalmic research institutes and contains refractive surgery centers, optometry centers, and corneal and ocular surface disease centers. It is equipped with professional instruments and can facilitate customized treatment for KC patients at different stages.

The inclusion criteria of clinical KC were as follows^7,19^: asymmetric bowtie pattern with or without skewed axes revealed by corneal topography and at least one KC clinical sign detected by slit-lamp examination (localized stromal thinning, Vogt’s striae, Fleischer’s ring, conical protrusion, or anterior stromal scar). Unilateral KC was considered if a patient had KC in one eye but did not meet the diagnostic criteria in the contralateral eye.

### Clinical data

Objective refraction including autorefraction (Topcon KR-800) and retinoscopy examination (scissor reflex sign), and subjective refraction, including phoropter (Topcon CV-5000) and trial lenses, were conducted in the subjects in the current study. A standard logarithmic visual acuity chart with a lightbox was used to obtain VA values. Clinical signs for KC were recorded. The best corrected distance visual acuity (CDVA) was converted to the logarithm of the minimum angle of resolution (logMAR) unit and used for further analysis.

Corneal topography and tomography were obtained through a Pentacam HR Imaging System (Oculus, Wetzlar, Germany), which uses the Scheimpflug imaging technique to present indices with acceptable accuracy and repeatability^20^. The following parameters were evaluated in the current study: the thinnest corneal thickness (TCT); mean, steep and the max keratometry (Km, Ks, Kmax); inferior superior (I-S) value; and Belin Ambrósio display (BAD). The corneal biomechanical parameters were measured through corneal visualization Scheimpflug technology (Corvis ST, Oculus 72100, Wetzlar, Germany). The acceptable repeatability of Corvis ST parameters has been described elsewhere in the published literature^21^. The following parameters were recorded in the current analysis: intraocular pressure (IOP), biomechanical corrected IOP (bIOP), stiffness parameter at the first applanation (SP-A1) and Corvis biomechanical index (CBI).

The slit-lamp examination, Pentacam and Corvis ST measurements were conducted by professional clinicians between 9:00 and 17:00. If the patients wore RGP lenses at their first visit, they were asked to stop wearing RGP lenses for two weeks and reexamined.

### Questionnaire survey

The demographic questionnaire and VF questionnaire survey were conducted by well-trained staff during face-to-face interviews at the clinic.

The questionnaire and survey were conducted when the patients first visited the hospital during the study period. For patients who had been diagnosed with KC before the study began, a check-up was performed via phone call, and the questionnaire was performed at their follow-up visit at our clinic. Age (enrollment age, KC onset age, and myopia onset age), education level (< high school, high school, > high school), residence (urban/rural), eye rubbing history, allergic disease history, onset condition, and type of vision correction before diagnosis were collected from the demographic questionnaire. A positive family history was defined as at least one first-degree relative diagnosed with KC. First-degree relatives (parents, siblings and offspring) of 142 KC patients had undergone examinations (slit-lamp examination, vision examination, Pentacam HR and Corvis ST measurements) to evaluate the positive family history. The allergic disease history of KC patients was defined as a previous diagnosis of allergic disease by a clinician. Wearing glasses, wearing rigid gas permeable (RGP) lenses, cross-linking (CXL) surgery and keratoplasty are the main management strategies for current KC patients.

The VFQ-25 consists of a base set of 25 vision-targeted questions representing 12 subscales: general health (1); general vision (1); mental health symptoms due to vision (4); ocular pain (2); difficulty with near-vision activities (3); difficulty with distance-vision activities (3); limitations in peripheral vision (1); limitations in social functioning due to vision (2); driving difficulties (3); color vision (1); role limitations due to vision (2); and dependency on others due to vision (3). It has been widely used to assess ocular conditions, such as cataracts, KC, glaucoma, macular degeneration, diabetic retinopathy, and CMV retinitis^22–24^. The methodology and validity of the VFQ-25 has been described previously^9,25^. In the present study, the VFQ-25 questionnaire was used to evaluate KC patients’ difficulty performing vision-related daily activities with their existing optical correction. The subscale scores, ranging from 0 (worst) to 100 (best), were calculated based on the scoring algorithm.

### Statistical analysis

Means ± standard deviations (SD) were calculated to describe qualitative data, and proportions were calculated to describe quantitative data. The Amsler–Krumeich (AK) classification was used to evaluate severity in all KC eyes^7^. The distributions of sex, residence and education level in KC patients were compared to those in the general population of Henan Province in 2019 using Chi-square tests (http://www.stats.gov.cn/tjsj/ndsj/2020/indexch.htm), and the ratios of people who wore glasses and RGP lenses were compared by McNemar tests. A general linear model or Chi-squared test was used to compare the demographic values, VF scores, clinical measurements, and treatments according to the disease severity. Spearman’s correlation analysis was used to determine the relationship between parameters and KC severity. A *P* value of < 0.05 (two-tailed) was considered statistically significant.

### Ethics approval and informed consent

This study was conducted according to the Declaration of Helsinki guidelines and approved by the Institutional Review Board of Henan Eye Hospital [ethical approval number: HNEECKY-2019 (5)]. Written informed consent was obtained from each patient or the legal guardian of subjects under the age of 18.

## Results

### Clinical measurements

**Figure 1:**
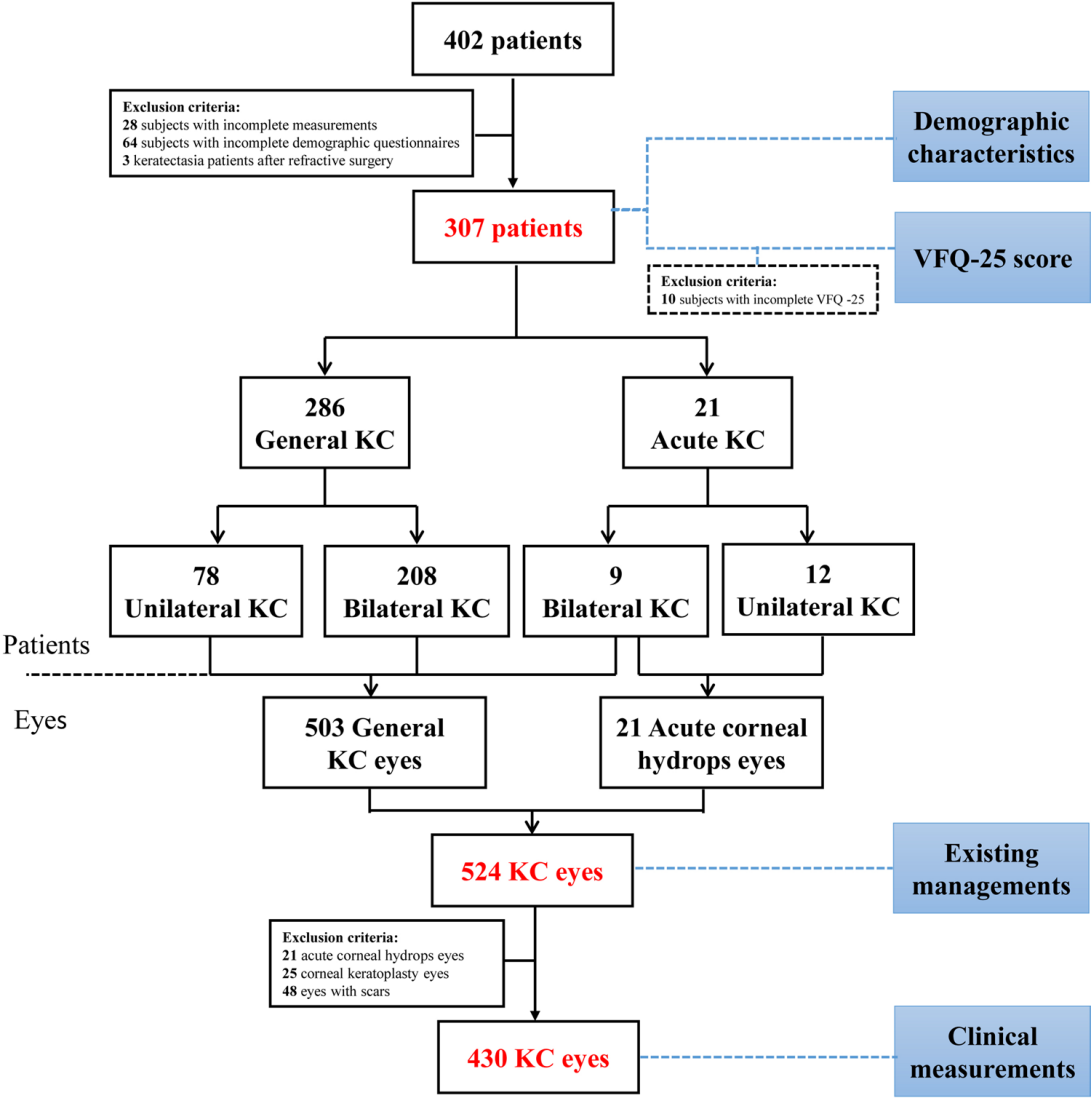
The inclusion and exclusion criteria in the current analysis.

A total of 402 KC patients visited the hospital during the study period. Among them, 28 subjects who had incomplete measurements, 64 participants who had incomplete demographic questionnaires, and 3 participants who developed keratectasia after refractive surgery were excluded. Finally, a total of 307 KC patients (217 bilateral and 90 unilateral) were included in the current analysis. Eyes with acute corneal hydrops (N = 21), corneal keratoplasty (N = 25) and scars (N = 48) affecting clinical data were excluded. Thus, a total of 430 KC eyes with qualified measurements were included to analyze corneal topography, tomography and biomechanical parameters (Fig. 1).

The mean CDVA was 0.52 ± 0.27. The scissor reflex was present in 94.04%, Vogt striae was present in 29.29%, Fleischer ring was present in 70.92%, Munson sign was present in 47.49% and corneal scarring was present in 12.76% of patients. Table 1 presents the Pentacam HR and Corvis ST measurements of the KC eyes. The mean TCT was 455.25 ± 48.67 μm, and the Km was 49.70 ± 5.98 D. The SP-A1 value for all KC patients was 69.40 ± 23.23 mmHg/mm.

**Table 1:** The clinical data of 430 KC eyes.

Variables	Mean ± SD	Range
TCT (µm)	455.25 ± 48.67	(237, 592)
Km (D)	49.70 ± 5.98	(39.8, 79.2)
Ks (D)	51.49 ± 6.47	(41.6, 84.2)
Kmax (D)	58.80 ± 10.48	(43.2, 93.9)
I–S value	5.09 ± 3.50	(− 5.39, 17.25)
BAD	10.82 ± 6.05	(2.68, 38.42)
IOP (mmHg)	13.07 ± 2.73	(5, 24)
bIOP (mmHg)	14.64 ± 2.51	(5.4, 24.9)
SP-A1 (mmHg/mm)	69.40 ± 23.23	(12.49, 135.18)
CBI	0.91 ± 0.24	(0, 1)

*TCT* thinnest corneal thickness, *Km* mean keratometry, *Ks* steep keratometry, *Kmax* the maximum keratometry, *IS-Value* inferior-superior value, *BAD* Belin Ambrósio display, *IOP* intraocular pressure, *bIOP* biomechanical corrected intraocular pressure, *SP-A1* stiffness parameter at the first applanation, *CBI* corvis biomechanical index.

### Demographic characteristics

Table 2 presents the demographic parameters of KC patients. The mean enrollment and diagnosis ages were 22.68 ± 6.50 and 20.98 ± 6.06 years, respectively. Males had a higher ratio of KC than females (*P* < 0.001), and patients living in rural areas had a higher rate than those living in urban areas (*P* = 0.039). The proportion of KC patients with a higher education (above high school) level was high (*P* < 0.001, Supplementary Table 1). In addition, 68.40% of the patients reported eye rubbing, 3.52% had a positive family history, and 12.70% had an allergic disease history. A total of 125 patients were diagnosed when preparing for/replacing the glasses at optometry clinics. Ninety-two (29.97%) patients had not received any eye care before diagnosis.

**Table 2:** Demographic characteristics of 307 patients.

Variables	N	Mean ± SD/(%)	Range
**Age (years)**			
Enrollment age	307	22.68 ± 6.50	(12, 54)
KC onset age	307	20.98 ± 6.06	(12, 46)
Myopia onset age	221^#^	13.97 ± 3.68	(6, 35)
**Enrollment age (years)**			
< 20	115	37.46	–
20–29	152	49.51	–
≥ 30	40	13.03	–
**Sex**			
Male	223	72.64	–
Female	84	27.36	–
**Educational level**			
< High school	52	16.94	–
High school	102	33.22	–
> High school	163	49.84	–
**Residence**			
Urban	145	47.23	–
Rural	162	52.77	–
**Onset condition**			
Preparing for/replacing glasses	125	40.72	–
Examination before refractive surgery	17	5.54	–
Physical examination	14	4.56	–
Direct hospital visit	126	41.04	–
Other	25	8.14	–
**Vision correction before diagnosis**			
None	92	29.97	–
Stealth/mostly stealth	12	3.91	–
Glasses/mostly glasses	203	66.12	–
**Eye rubbing**	210	68.40	–
**Family history of KC**	5*	3.52	-
**Allergic disease history**	39	12.70	–

^#^A total of 221 KC patients had a history of myopia before diagnosis.

*Family members of 142 KC patients were examined.

### Scores of the VFQ-25

**Figure 2:**
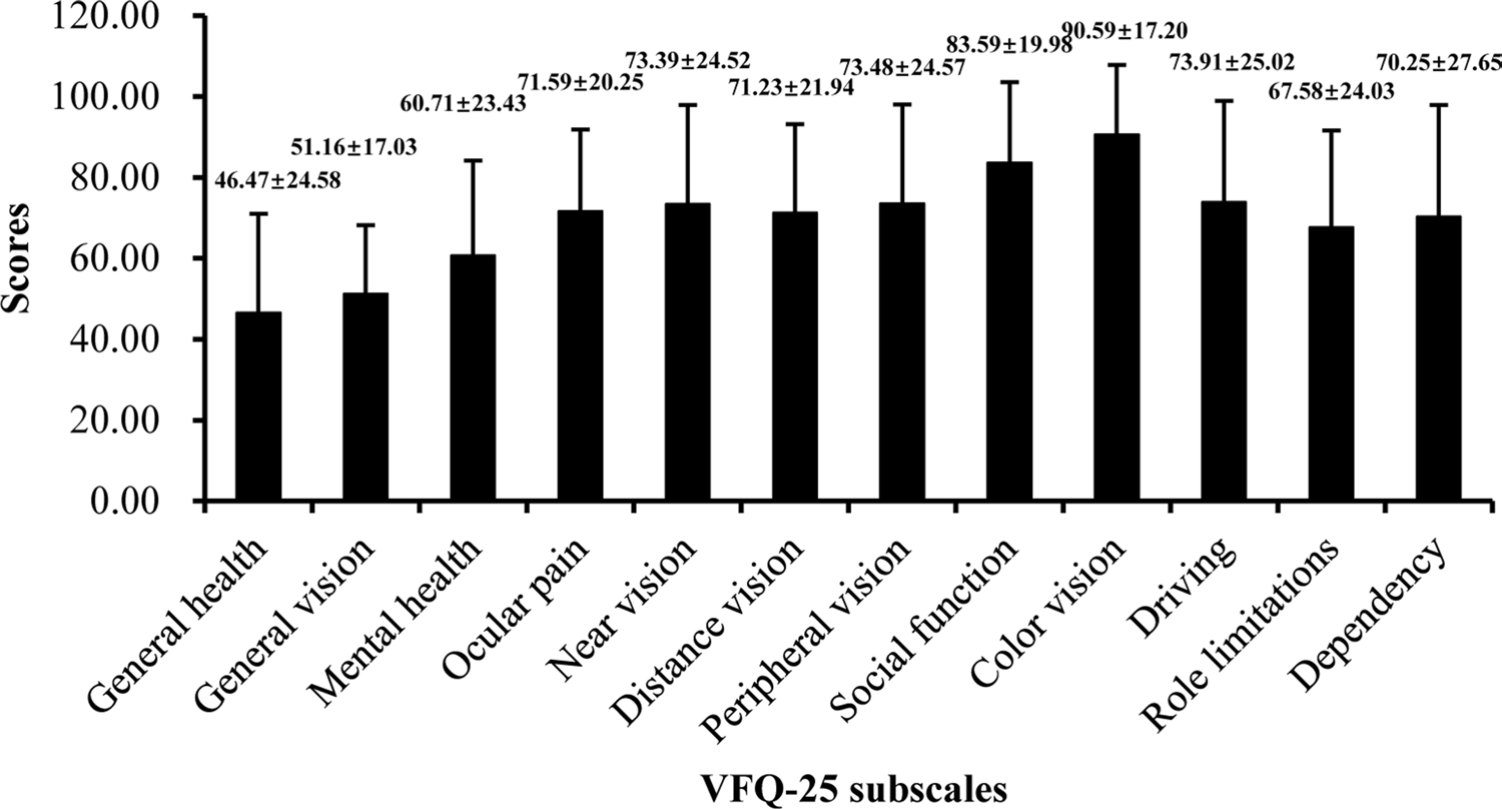
Averaging items to generate VFQ-25 subscales in KC patients.

In total, 297 of the 307 KC patients who were included the VF score evaluation had complete VF information. The total VF score in KC patients was 69.35 ± 15.25. The scores of the 12 subscales are presented in Fig. 2. The scores of the 12 subscales ranged from 46.47 ± 24.58 (general health) to 90.59 ± 17.20 (color vision).

### Existing management strategies

**Figure 3:**
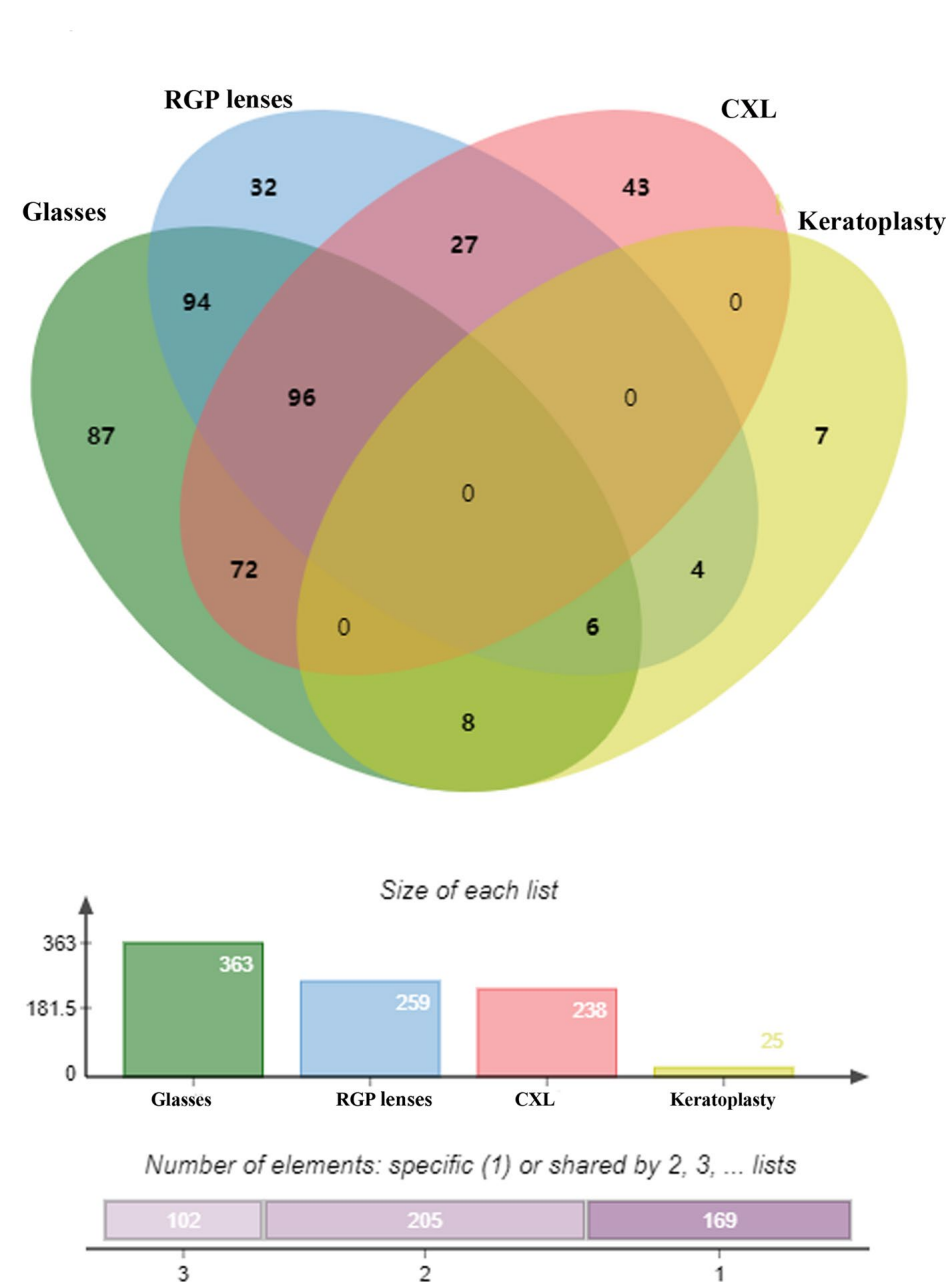
Existing management strategies in 524 KC eyes (48 KC eyes received no treatment). *CXL* cross-linking; RGP, rigid gas permeable.

Figure 3 shows the existing management strategies for 524 KC eyes. A total of 363 KC eyes were corrected with glasses, 259 KC eyes were corrected with RGP lenses, 238 KC eyes had been treated with CXL surgery, 25 KC eyes had undergone keratoplasty, and 48 KC eyes had not received treatment (9.16%). More people wore glasses than RGP lenses (glasses vs RGP lenses: 69.71% vs 49.19%, *P* < 0.001).

### Correlation between parameters and disease severity

**Figure 4:**
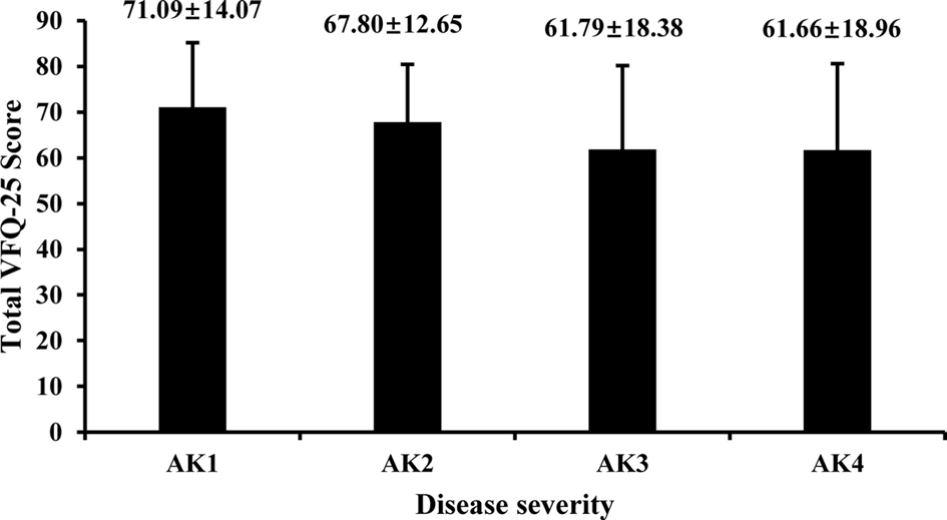
The total VFQ-25 score according to KC disease severity (F = 4.055, *P* = 0.008).

Table 3 presents the clinical and demographic data according to AK classification. The percentages of AK1, AK2, AK3, and AK4 eyes were 23.66%, 26.15%, 20.80% and 29.39%, respectively. The results showed that TCT and SP-A1 values were negatively associated with KC severity, and the Km, Ks and Kmax values; the percent of RGP lenses; and keratoplasty were positively associated with KC severity (all *P* < 0.05). The eyes with better VA in 217 bilateral KC patients were included in the correlation analysis between total VF score and disease severity. The total VF of the eye with better VA decreased as the severity increased (r = − 0.21, *P* = 0.002, Fig. 4).

**Table 3:** The clinical, demographic, and behavioral data according to AK classification.

Parameters	AK1 (N = 124)	AK2 (N = 137)	AK3 (N = 109)	AK4 (N = 154)	F/χ^2^	*P*^*#*^	r	P*
**Clinical data**								
TCT (µm)	482.92 ± 33.92 (419, 592)	464.83 ± 33.92 (395, 568)	450.68 ± 40.16 (369, 563)	402.03 ± 55.30 (237, 534)	65.442	< 0.001	− 0.52	< 0.001
Km (D)	44.93 ± 1.62 (40.9, 47.9)	47.86 ± 2.83 (39.8, 52.7)	50.37 ± 3.28 (43.0, 54.9)	58.83 ± 6.12 (45.1, 77.0)	248.214	< 0.001	0.76	< 0.001
Ks (D)	46.14 ± 2.05 (41.6, 50.9)	49.97 ± 3.53 (43.2,58.6)	52.39 ± 3.74 (43.3, 59.8)	61.30 ± 6.85 (46.7, 84.2)	215.330	< 0.001	0.74	< 0.001
Kmax (D)	50.43 ± 4.69 (43.2, 65.6)	56.73 ± 6.77 (43.8, 93.9)	60.65 ± 7.61 (43.8, 78.5)	73.46 ± 10.13 (48.1, 92.5)	160.454	< 0.001	0.69	< 0.001
SP-A1 (mmHg/mm)	84.90 ± 18.45 (38.22, 128.83)	71.57 ± 19.93 (38.58, 135.18)	63.68 ± 20.39 (24.15, 123.75)	48.52 ± 20.78 (12.49, 131.18)	54.883	< 0.001	− 0.55	< 0.001
**Demographic data**								
Enrollment age	23.02 ± 7.09 (12, 46)	22.69 ± 6.36 (12, 46)	22.25 ± 5.18 (13, 40)	23.48 ± 7.49 (13, 54)	0.796	0.496	0.02	0.665
Sex (M/F)	86/38	94/43	75/34	116/38	2.177	0.537	− 0.05	0.254
**Behavioral data**								
Eye rubbing (no/yes)	47/77	47/90	38/71	47/106	1.601	0.659	0.05	0.238
Glasses (no/yes)	38/86	43/94	21/88	58/96	13.389	0.004	− 0.04	0.413
RGP lenses (no/yes)	80/44	68/69	41/68	77/77	12.939	0.005	0.11	0.010
CXL surgery (no/yes)	84/40	59/78	42/67	101/53	27.927	< 0.001	− 0.01	0.985
Keratoplasty (no/yes)	124/0	137/0	109/0	129/25	16.224	0.001	0.28	< 0.001

*TCT* thinnest corneal thickness, *Km* mean keratometry, *Ks* steep keratometry, *Kmax* the maximum keratometry, *SP-A1* stiffness parameter at the first applanation, *RGP* rigid gas permeable, *CXL* cross-linking.

*P*^*#*^: the statistical value of the general linear model or Chi-squared test, P*: the statistical value of Spearman’s correlation analysis.

## Discussion

KC is a progressive disease that leads to the development of corneal steepening, thinning, and asymmetric distortion in the apical zone of the cornea^1^. In this hospital-based study, we comprehensively evaluated various features, including clinical data, demographic factors, and VF questionnaire data of 307 KC patients in Central China.

KC is a corneal protrusion disorder, and Pentacam HR parameters have been demonstrated effective in diagnosing KC and evaluating its progression^8,26^. The TCT values in the present study were higher, and the Km values were lower than those reported by Naderan et al.^27^. Abnormal biomechanical parameters are observed at an early stage before tomographic changes and clinical symptoms in KC^28^. Our previous study showed that eyes with KC had lower values of SP-A1 than normal eyes, and the value of SP-A1 was consistent with the current results^21^. In addition, the current study showed that the Km value increased and SP-A1 value decreased as disease severity decreased, indicating that the cornea became steeper and corneal stiffness became weaker as disease severity decreased^29^. Slit-lamp examination is necessary for evaluating eyes with KC. The percentages of Vogt striae, Fleischer ring, and corneal scarring in the present study were consistent with those reported by Khor et al.^5^, but lower than those reported in the Collaborative Longitudinal Evaluation of KC (CLEK) study^14^.

KC presents at puberty and progresses until the third or fourth decade of life^8^. The mean age at diagnosis was 20.98 years in our study, which was lower than that reported in previous studies^30–32^. The mean age of KC patients from the CLEK was 39.29 ± 10.90 years^30^. Tufts et al.^31^ reported that the age at KC diagnosis was 22.4 years in males and 23.3 years in females. Hwang et al.^32^ further reported that the median age at diagnosis was 29 years in 13,343 KC patients in South Korea. The ratio of KC patients aged < 20 years was lower in our study than in the study by Abu Ameerh et al.^33^. In addition, KC was diagnosed in a higher proportion of males than females, which was consistent with previous findings^5,15^. The proportion of KC patients with a higher education level was high, which is similar to the results reported in a study conducted in Denmark^15^. Differences in demographic data may be attributed to the anatomic, physiologic, hormonal, genetic and lifestyle habits as well as personality factor differences^34^.

A recent study reported that eye rubbing, disease history, and family history of KC are critical factors associated with the incidence of KC^2^. Eye rubbing, a common physiological response to a sensation of eye discomfort, can be induced by fatigue, dust or allergen exposure, and contact lenses^35^. The present study showed that 68.40% of KC patients had an eye rubbing history, and this proportion was within the range of 44.8–83% reported in a previous study^36^. The association between eye rubbing and KC may be related to keratocyte density and IOP^36^. However, the correlation between IOP and eye rubbing was not statistically significant in the current study (r = − 0.075, *P* = 0.091). Furthermore, no significant difference was found between eye rubbing and KC severity. Further studies with a control group to explore the association between eye rubbing and KC incidence are needed. Allergic conditions, as one of the primary triggers that stimulate eye rubbing, were also found to be associated with KC^37^. Approximately 25% of KC patients in India had systemic or ocular allergies^38^, and 34.4% of KC patients in Israel were reported to have allergies^39^, and 17.6% of KC patients had environmental allergies in a study by Nemet et al.^40^. The proportion of allergic diseases in the current study was lower than that in other studies, which may be explained by the differences in physiologic, hormonal, and genetic factors as well as lifestyle habits^38–40^.

A positive family history of KC is a strong indication of a genetic predisposition, and the ratio of patients with a family history of KC was inconsistent with previous literatures^4,5,14,41–44^. We found that the ratio of positive first-degree family history was 3.52%, which was lower than the proportions reported by Gordon-Shaag et al. (26%)^44^, Millodot et al. (21.7%)^42^, Naderan et al. (19.5%)^4^, Szczotka-Flynn et al. (17.8%)^41^, Karimian et al. (14%)^43^, Zadnik et al. (13.5%)^14^ and Khor et al. (4.3%)^5^. Lower response rates in first-degree relatives with KC and different criteria for determining a positive family history have caused the percentage differences between these studies^45^. Data from a large number of family members’ clinical examinations were being collected in the present study, and further analysis will be conducted in the future.

The present study showed that patients in rural areas had a higher incidence of KC than those from urban areas, and 125 patients of 307 patients were diagnosed with eye abnormalities, including a rapidly increase in spherical equivalent or/and astigmatism value, or/and a decrease in CDVA value, at optometry clinics. Furthermore, the percentage of AK4 eyes among 524 KC eyes was 29.39%, and one-third of 307 KC patients had not received eye care before diagnosis. This phenomenon may be attributed to the medical insurance system in China and the demographic data of participants. The Chinese health system is a basic medical insurance system (BMIS), which is an important part of the medical security system with Chinese characteristics^46^. The cost of keratoplasty is covered by insurance. However, the costs of KC diagnostic examinations and management strategies (such as glasses, RGP lenses, and CXL surgery) are not covered by the insurance. In addition, regular eye examinations are not yet available to all residents in China, and patients often visit hospitals only if they have symptoms. Most patients might seek the help from optometry clinics, which are not equipped with diagnostic tools, leading to missed diagnosis of mild KC^33^. Patients usually visit the hospital when they are unable to obtain adequate care at optometry clinics. The rural population in Henan Province is relatively larger, and the per capita disposable income in Henan Province is lower than those in Beijing (northern China) and Shanghai (eastern China)^16,17^. The percentage of patients with KC in the capital district is much higher because the diagnosis is not covered by health insurance. The results provide a reference for understanding the characteristics of KC patients in China, and a multicenter study combining the data from local hospitals and other regions needs to be conducted in the future.

The unpredictable nature of visual disturbance caused by KC affects the daily activities and emotional well-being of patients^47^. The findings reported by the CLEK study have shown that the scores of the 12 subscales were higher than those reported in the present study^12^. The total VF score in current KC patients was lower than those in KC patients from France^13^, Iran^10^, and Istanbul^48^, whereas it was quite similar to those in KC patients from Japan^49^ and Diyarbakir^50^. Several studies have indicated that VF is sensitive enough to detect KC at an early stage because of early visual disturbances induced by vision loss and irregular astigmatism^25,49^. In addition, the current study found that the total VF score in the eye with better VA was negatively associated with the disease severity, which is consistent with previous studies^12,51^. However, no significant association was found between VF scores and KC grades in a study by Tatematsu-Ogawa et al.^49^, in which Japanese KC patients had a higher mean age and were divided into three groups according to CDVA value. The differences in associations might be explained by the ethnic differences and the types of KC classification; AK classification was used in the current research. The VFQ-25 measurements, which provide information about KC patients, are beneficial to clinicians, and more studies should be conducted in the future.

The management of KC, as a progressive disease, varies with disease severity. At an early stage, treatment is usually achieved with glasses. As the disease progresses, astigmatism worsens, and glasses cannot correct it; at that point, RGP lenses become the principal therapeutic option^52^. The present study’s results with regard to the proportion of KC patients with RGP lenses was similar to that reported by Agrawal (40%)^38^ but were lower than that reported by Owens et al. (83%)^53^ and Shneor et al. (78.7%)^39^ and that reported in the CLEK study (75%)^14^. The difference may be attributed to the various medical systems and the availability, intolerance, and the affordability of RGP lenses^38^. In addition, the percentage of patients wearing glasses was higher than that of patients wearing RGP lenses, and the number of patients wearing RGP lenses increased as the severity of KC increased. This might be attributed to the high cost of RGP lenses and the relatively low economic status of subjects in Central China, which is different from other regions of China and other countries. Significant challenges in the management of progressive KC are to improve vision and arrest the progression of the disease^54^. The effectiveness of CXL surgery in arresting disease progression has been reported in several studies^55,56^. In advanced to severe stages, when glasses or RGP lenses cannot improve vision, keratoplasty is performed^39^. The present study showed that the percentage of patients who underwent keratoplasty increased with increasing KC severity. However, keratoplasty complications, such as rejection, a fixed dilated pupil, postoperative astigmatism, and recurrence of KC, affect the application of keratoplasty^1^. The treatments available for KC have been demonstrated effective, and the critical consideration is to select the best treatment for the KC patient.

The current study was based on a hospital-based population. Although the hospital is a tertiary hospital with trained and experienced ophthalmologists, the study does not fully represent the whole population of China. This hospital-based study analyzed the clinical features of the disease and evaluated the effects of therapeutic measures^57^. However, some bias still exists that may affect the research results. In addition, certain limitations in the current study should be noted. This is a descriptive study that relied on clinical records. Some patients had been diagnosed with KC for over 5 years, and the demographic and onset data that were obtained through the questionnaire may be biased. In addition, the study investigated KC patient characteristics, with no control group, affecting the extrapolation of the results. A multicenter and large sample study on KC is needed, and the relevant results need to be validated further in the general population of China in future studies.

In conclusion, the present study comprehensively described various associated features in 307 KC patients in Central China, combining clinical data, demographic data and VF questionnaire data. The results reported here may help us better understand the characteristics of KC patients in China.

## Supplementary Information

Supplementary Table.

## Data Availability

All relevant data are included in the papers and its Supporting Information files. Contact to Dr. Shengwei Ren (shengweiren1984@163.com) for additional information regarding data access.
