# Supplementary material for: A hospital-based study on clinical data, demographic data and visual function of keratoconus patients in Central China
Source: Sci Rep. 2021 Apr 6;11:7559. doi: 10.1038/s41598-021-87291-y (PMC8024332; doi:10.1038/s41598-021-87291-y)
Supplement: Supplementary file 1 — Supplementary Table. [file 41598_2021_87291_MOESM1_ESM.pdf]

A hospital-based study on clinical data, demographic data and visual function of keratoconus patients in Central China

Running Head

Characters of Chinese keratoconus

Authors:

Kaili Yang †, Liyan Xu †, Qi Fan, Yuwei Gu, Bo Zhang, Feiying Meng, Dongqing Zhao, Chenjiu Pang, Shengwei Ren *

Authors affiliations:

Henan Provincial People's Hospital, Henan Eye Hospital, Henan Eye Institute, People's Hospital of Zhengzhou University, Henan University People's Hospital, Zhengzhou, 450003, China.

†Contributed equally to this work.

*** Correspondence author**

Shengwei Ren

Henan Provincial People's Hospital

Henan Eye Hospital, Henan Eye Institute

People's Hospital of Zhengzhou University

Henan University People's Hospital

7 Weiwu Road, Zhengzhou, 450003, Henan, *PR* China

Phone: +86 371 65580908

E-mail: shengweiren1984@163.com

Supplementary Table 1 Comparison of gender, residence and education level between KC patients and general population of Henan Province.

**Parameter**	**KC (N=307)**	**General population (N=96400000)**	χ^2	***P***
**Gender, n(%)**			57.455	<0.001
Male	223 (72.64)	48850000 (50.67)		
Female	84 (27.36)	47750000 (49.33)		
**Residence, n(%)**			4.263	0.039
Urban	145 (47.23)	51290000 (53.21)		
Rural	162 (52.77)	45110000 (46.79)		
**Educational level, n(%)***			642.90	<0.001
<High school	52 (16.94)	49040 (70.88)		
High school	102 (33.22)	12716 (18.38)		
> High school	163 (49.84)	7433 (10.74)		

*the total number people of education level in general population of Henan Province is based on 2019 National Population Sampling Survey
